# Macrophage–Bacteria Interactions—A Lipid-Centric Relationship

**DOI:** 10.3389/fimmu.2017.01836

**Published:** 2017-12-20

**Authors:** Ooiean Teng, Candice Ke En Ang, Xue Li Guan

**Affiliations:** ^1^Lee Kong Chian School of Medicine, Nanyang Technological University, Singapore, Singapore

**Keywords:** lipids, metabolism, macrophage, intracellular bacteria, infection, immunity, tuberculosis, salmonellosis

## Abstract

Macrophages are professional phagocytes at the front line of immune defenses against foreign bodies and microbial pathogens. Various bacteria, which are responsible for deadly diseases including tuberculosis and salmonellosis, are capable of hijacking this important immune cell type and thrive intracellularly, either in the cytoplasm or in specialized vacuoles. Tight regulation of cellular metabolism is critical in shaping the macrophage polarization states and immune functions. Lipids, besides being the bulk component of biological membranes, serve as energy sources as well as signaling molecules during infection and inflammation. With the advent of systems-scale analyses of genes, transcripts, proteins, and metabolites, in combination with classical biology, it is increasingly evident that macrophages undergo extensive lipid remodeling during activation and infection. Each bacterium species has evolved its own tactics to manipulate host metabolism toward its own advantage. Furthermore, modulation of host lipid metabolism affects disease susceptibility and outcome of infections, highlighting the critical roles of lipids in infectious diseases. Here, we will review the emerging roles of lipids in the complex host–pathogen relationship and discuss recent methodologies employed to probe these versatile metabolites during the infection process. An improved understanding of the lipid-centric nature of infections can lead to the identification of the Achilles’ heel of the pathogens and host-directed targets for therapeutic interventions. Currently, lipid-moderating drugs are clinically available for a range of non-communicable diseases, which we anticipate can potentially be tapped into for various infections.

## Introduction

Macrophages play a key role as the front line of host defenses against foreign bodies. Complex scavenger receptors (SR), pattern recognition receptors, and other signaling receptors expressed by macrophages make them professional phagocytes and antigen-presenting cells. They are highly specialized in engulfment and digestion of the invading pathogen, followed by presentation of antigens to T cells. Under normal circumstances, cytokines and chemokines are secreted by macrophages once pathogens are detected, to recruit more immune cells to the area of infection for restriction of pathogen invasion. Traditionally, macrophages have been classified as inflammatory macrophages (also known as classically activated or M1 macrophages) or anti-inflammatory macrophages (also known as alternatively activated or M2 macrophages), based on their polarization states and physiological features (1, 2). However, it is increasingly appreciated that these cells display remarkable plasticity and their identities may be far more complex, as described in a comprehensive review by Mosser and Edwards (3). The inflammatory and anti-inflammatory responses of macrophages are tightly regulated at different infection stages and are pathogen-specific. Disturbance in this equilibrium will lead to excessive inflammation, or failure to activate the immune response, and this is often exploited by pathogens through the hijack of host signaling mechanisms to evade clearance by professional phagocytes. In fact, various intracellular pathogens have evolved strategies to reside and thrive in macrophages, despite the bactericidal capacity of these host cells. For instance, *Mycobacterium tuberculosis, Legionella pneumophila*, and *Salmonella enterica* serovar Typhimurium enter macrophages and persist in vacuolar compartments by modifying the vacuolar maturation processes, while others, including *Listeria monocytogenes, Shigella flexneri, Rickettsia rickettsii*, and *Mycobacterium marinum* escape the phagosome and replicate in the cytosol. *M. tuberculosis* and *Mycobacterium leprae* can also escape the phagolysosomal compartment into the cytosol, and this process is mediated by secreted proteins, including culture filtrate protein 10 and early secreted antigenic target 6 kDa (ESAT-6) (4). From the host perspective, it is well established that macrophages make use of soluble proteins for communication with other immune cells for initiation of complex signaling cascades during the infection process. In addition, it is increasingly evident that lipids play an equally important role in macrophage functions, influencing the outcome of infections (5–9).

Lipids are fundamental building blocks of cells and play pivotal roles in diverse biological processes. They are key structural components for cellular membranes. With their hydrophobic and amphipathic properties, lipids are able to form a barrier between cells and their environments, as well as within the cells to produce distinct organelles. These cellular membranes have the ability to mediate cell–cell and intracellular communication by budding, fission, and fusion. Lipids also serve as energy stores in eukaryotic cells, in the form of triglyceride esters and steryl esters in lipid droplets. In addition, they play a key role as first and second messengers in signaling cascades. The functions of membrane lipids as well as the lipid composition of an average mammalian plasma membrane have been thoroughly examined and reviewed previously (7, 10, 11).

**Figure 1:**
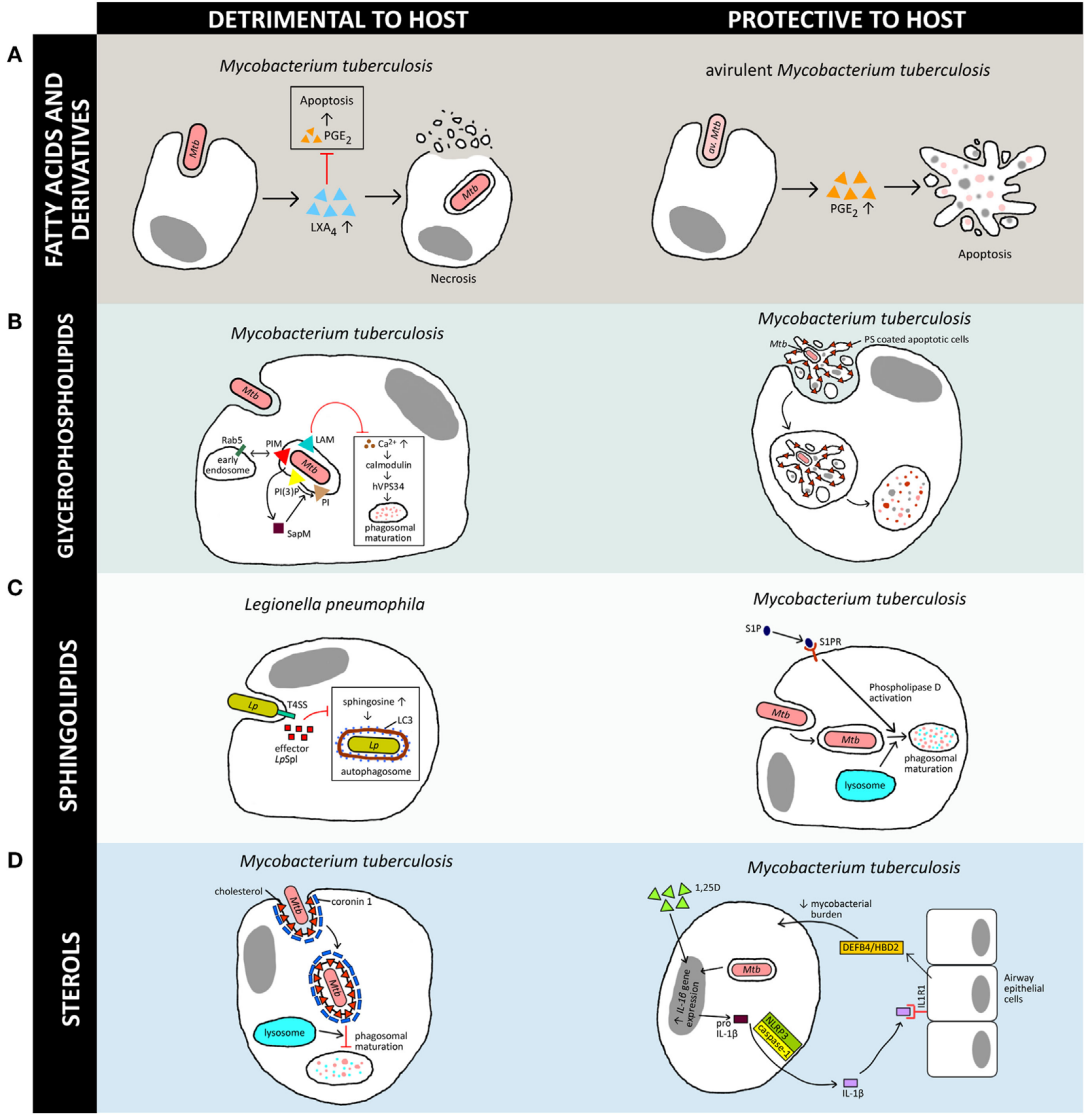
Versatility of lipids in generation of host immune responses against various intracellular pathogens. The schematic diagram illustrates a simplified overview of how the four host-derived lipid classes discussed in this review can be a double-edged sword, either being exploited by the pathogen for its own survival or aiding the host in clearance of the bacteria. Note that there are multiple lipids involved in host–pathogen interactions but only a few representative examples are shown here, and further details can be found in the original works. [**(A)**, left] Induction of LXA_4_ by virulent *M. tuberculosis* (*Mtb*) inhibits PGE_2_ signaling and promotes necrosis in macrophages (18), whereas [**(A)**, right] induction of PGE_2_ secretion by avirulent *M. tuberculosis* (av. *Mtb*)-infected macrophages leads to apoptosis and protects against mitochondrial inner membrane damage (19). [**(B)**, left] *Mtb* manipulates host phosphoinositides metabolism to promote their survival in macrophages *via* inhibition of phagosomal maturation. Mycobacterial phosphatidylinositol mannoside (PIM) stimulates early endosomal fusion by recruiting Rab5. Inhibition of Ca^2+^ increase by *Mycobacterium* lipoarabinomannan (LAM) further blocks phagosomal maturation as Ca^2+^ is required for calmodulin phosphatidylinositol 3-kinase hVPS34 signaling cascade activation. SapM secreted by *Mycobacterium* inhibits phagosomal-late endosome fusion by hydrolyzing phosphatidylinositol 3-phosphate (20–23). However, [**(B)**, right] redistribution of phosphatidylserine (PS) during apoptosis leads to efferocytosis and restricts the growth of *Mtb* (24). [**(C)**, left] Effector *Lp*Spl from *Legionella pneumophila* (*Lp*) mimics host sphingosine-1-phosphate (S1P) lyase and prevents an increase in sphingosine levels in infected macrophages, inhibiting autophagy (25). On the other hand, [**(C)**, right] S1P is essential for bacterial clearance as it promotes acidification of *Mycobacterium*-containing phagosomes *via* phospholipase D activation, which leads to phagosomal maturation and killing of *Mtb* (26). [**(D)**, left] Accumulation of cholesterol at the *Mtb* uptake site recruits coronin 1 protein, which inhibits phagosomal maturation (27). [**(D)**, right] The active metabolite of vitamin D (1,25D) controls *Mtb* infection *via* macrophage–epithelial paracrine signaling. IL-1β secreted through NLRP3/caspase-1 inflammasome signaling cascade stimulates epithelial cells to produce antimicrobial peptide DEFB4/HBD2, which reduces mycobacterial burden in macrophages (28). In this schematic diagram, triangles represent lipids whereas squares represent proteins.

In the context of macrophage–intracellular bacteria interactions, the plasma membrane of immune cells will be the first barrier the invading pathogen encounters. To successfully infect the host, pathogens often evolve various strategies to target the lipid-enriched plasma membrane for entry and exit, or to hijack host lipid metabolism to promote their survival (12–14). By contrast, lipids can also provide protection to the host during microbial infections (15), demonstrating that lipids are functionally double-edged swords. Here, we review the confounding nature of lipids in the intimate macrophage–pathogen relationship (Figure 1), with a focus on intracellular bacteria, as well as recent techniques employed to probe the dynamics of lipid metabolism during infections. It should be noted that this is not exhaustive as many other pathogens, including eukaryotic parasites, viruses, and fungal pathogens are capable of manipulating host lipid metabolism as part of their survival strategies (8, 9, 16, 17).

## Dynamic Lipid Remodeling During Macrophage Polarization

Macrophages undergo polarization when they encounter foreign bodies, and numerous lines of evidence point toward dynamic lipid remodeling during this defense process. Transcriptional profiling of human macrophage polarization in an *in vitro* experimental model revealed striking enrichment of genes involved in lipid metabolism as one of the most overrepresented categories of differentially modulated transcripts (29). For M1 macrophages, besides exhibiting preference for aerobic glycolysis, upregulation of cyclooxygenase (COX)-2 and downregulation of COX-1, leukotriene A4 hydrolase, thromboxane A synthase 1, and arachidonate 5-lipoxygenase (5-LO) were observed. On the other hand, M2 macrophages are skewed toward fatty acid oxidation and have upregulated expression of COX-1 and arachidonate 15-lipoxygenase (15-LO) (29–31). Interestingly, opposing regulation of sphingosine and ceramide kinases was observed in both M1 and M2 macrophages (29), highlighting the contrasting nature of lipid metabolism in these distinct macrophage populations.

Receptors involved in binding diverse lipid metabolism products, such as peroxisome proliferator-activated receptor α, β/δ and γ, liver X receptor α and β, CD36, and SR class A-I/II, have also been linked to the polarization of M2 macrophages (32–34). These receptors internalize oxidized and modified lipoproteins and are critical orchestrators of macrophage cholesterol and fatty acid homeostasis (33, 34). M2 macrophages increase oxidized low-density-lipoproteins cholesterol uptake but have similar cholesterol efflux as compared with M1 macrophages. This difference in cholesterol handling leads to increased cholesterol deposition in M2 macrophages (32). It has also been shown that the expression of SR such as CD36 or SR-A1 on M2 macrophages induces endocytosis of triglyceride-rich lipoproteins and generates fatty acids for β-oxidation (32, 35). This aspect of macrophage lipid metabolism is in fact, closely associated with the pathogenesis of atherosclerosis, and the burgeoning field of immunometabolism highlights the critical roles of the cross talk between metabolism and immunity (36).

The dynamics of lipid metabolism during polarization have been further demonstrated at the metabolite level in various studies (37, 38). A shift in the degree of saturation of fatty acids towards monounsaturated fatty acids was observed during human monocyte differentiation to M2 macrophages using macrophage colony stimulating factor (M-CSF), which is in agreement with the induction of sterol regulatory element-binding transcription factor 1c regulated genes, fatty acid synthase, elongation of very long-chain fatty acid-like family member 6 (ELOVL6), and stearoyl-CoA desaturase (37). These observations on the fatty acid composition of glycerophospholipids were further corroborated in a recent study by Zhang and coworkers (38). In addition, an increase in phosphatidylcholine and phosphatidylethanolamine, and a decrease in cholesterol, were observed during macrophage differentiation. Employing a combination of lipidomics, transcriptomics, and pharmacological and genetic manipulations, the direct connection between fatty acid and glycerophospholipid synthesis during the differentiation process was addressed. Furthermore, perturbation of macrophage lipid metabolism was also found to affect the ultrastructural and phagocytic properties of these immune cells (37). These results corroborate earlier findings on the effects of fatty acid unsaturation on macrophage phagocytic activities (39).

Overall, these studies suggest that the polarization of macrophages *in vitro* is intimately linked to lipid metabolism, and perturbations of host lipid metabolism will affect immune and cellular functions. However, it should be noted that the spectrum of macrophage populations *in vivo* is more complex (3). This warrants more detailed analyses to determine the exact functions of lipids in the distinct populations and is dependent on environmental cues. We will next examine how macrophage lipids play critical functional roles in the intimate host–microbe relationship.

## Host Lipids—The Protagonist and the Antagonist in the Macrophage–Microbe Relationship

### Fatty Acids and Derivatives

In macrophages, fatty acids can be synthesized *de novo* or be taken up by cells through lipolysis of triglyceride-rich lipoproteins particles such as low-density lipoproteins and very-low-density lipoproteins. This is achieved by the expression of SR such as CD36 (40) or SR-B1 (41). The function of fatty acids in various cellular components has a long history and has been extensively reviewed (42). Being the major component of triglycerides, glycerophospholipids, and other complex lipids, fatty acids play pivotal roles as membrane constituents and energy sources, and partake in regulation of signaling pathways as well as cell and tissue metabolism (43). Fatty acids are also substantially found in bacterial cell membranes (44) and serve as precursors for membrane biogenesis. Persistence of *M. tuberculosis*, an intracellular bacterium and the causative agent of tuberculosis, in mice, is facilitated by isocitrate lyase, an enzyme essential for fatty acid metabolism (45). This highlights the role of fatty acids for maintenance of mycobacterial survival during chronic infections. In fact, besides *de novo* synthesis, pathogens including *M. tuberculosis* have evolved multiple strategies to utilize fatty acids derived from their hosts. *M. tuberculosis* imports fatty acids from host triglycerides for synthesis of its own lipid inclusions and acquisition of dormancy traits in macrophages (46, 47). The mycobacterial protein, LucA, was found to form a complex with Mce1 and Mce4 fatty acid transporters to facilitate cholesterol and fatty acid uptake in infected macrophages (48). In dormant *M. tuberculosis*, the accumulation of triglycerides is modulated by fatty acid CoA ligase 6 (49), and during nutrient starvation, the bacterium is capable of hydrolyzing these stored triglycerides, owing to its lipase activity (50).

Interestingly, many intracellular pathogens also induce the formation of lipid droplets in the hosts during infections. This has previously been reviewed by Saka and Valdivia (51) and more recently by Barisch and Soldati (52). Besides serving as an energy reservoir, triglycerides found in lipid droplets are also producers of the mediator lipids, eicosanoids, in mammalian cells. Eicosanoids, which are oxidation products of arachidonic acid and other polyunsaturated fatty acids, have assumed a key role as mediators of the immune response during infections (53, 54), and as outlined above, have been shown to be differentially regulated during macrophage polarization. Broadly, it is noted that the effects of eicosanoids in host–pathogen interactions can be classified as pro-inflammatory (54) or anti-inflammatory (55), and they are closely interlinked with their protein counterparts, the cytokines, for orchestration of immune responses. Eicosanoids such as prostaglandin E2 (PGE_2_) and lipoxin A4 (LXA_4_) are highly active lipid mediators commonly described in bacterial infections. PGE_2_ was found to be upregulated by various invasive bacteria, including *Mycobacterium bovis* bacillus Calmette–Guérin (BCG) (19), *L. monocytogenes, Yersinia enterocolitica, Shigella dysenteriae*, and the enteroinvasive *Escherichia coli* (56). These mediator lipids can benefit the host through various mechanisms. In avirulent *M. tuberculosis* infections, PGE_2_ has been found to modulate host cell death pathways through the EP2 receptor, which promotes protection of the host against mitochondrial inner membrane perturbation and restricting the spread of bacterium caused by necrosis (57) (Figure 1A, right panel). Interestingly, PGE_2_ is found to be involved in the repair of host plasma membranes by regulating synaptotagmin 7, the calcium sensing protein involved in lysosome-mediated membrane repair (58). Resealing of membrane lesions is crucial for preventing necrosis and promoting apoptosis, which benefits the host by increasing bacterial clearance.

By contrast, eicosanoids may also bring about detrimental effects to the host, which is to the pathogen’s advantage. PGE_2_, earlier discussed to be beneficial in mycobacterial infections, can compromise host immunity through inhibition of macrophage maturation (59) and reduction in the release of NAPDH oxidase (60). Upregulation of PGE_2_ during infections is indeed beneficial for the survival of various pathogens, including *Salmonella enterica* (61) and *Burkholderia pseudomallei* (62). During *S. enterica* serovar Typhimurium infections, the *Salmonella* pathogenicity island (SPI)-2-encoded SpiC protein activates the ERK1/2 signaling pathway, leading to induction of COX-2 and increased production of PGE_2_ levels (61). PGE_2_ impairs killing of *Salmonella* species in macrophages by inducing IL-10 expression *via* the protein kinase A pathway (63). During *B. pseudomallei* infection in macrophages, PGE_2_ mRNA was rapidly upregulated by over 400-fold at as early as 2h postinfection and promoted *B. pseudomallei* intracellular survival (62). The suppression of the bactericidal activity of macrophages was associated with decreased nitric oxide production through enhancing the expression of enzyme arginase 2 (62).

LXA_4_ is another prominent mediator with anti-inflammatory effects (64) generated by 5-LO and 15-LO activities (65). Upregulation of LXA_4_ levels during mycobacterial infections can lead to inhibition of PGE_2_, and consequently mitochondrial inner membrane perturbation and macrophage necrosis (57). Unlike PGE_2_ that protects the host during mycobacterial infections, 5-LO promotes the growth of the bacterium *in vivo* (66). Virulent *M. tuberculosis* induces LXA_4_ and inhibits PGE_2_ production (18). By inhibiting the production of PGE_2_, LXA_4_ impairs apoptotic signaling (67) and promotes necrosis of infected macrophages (18) (Figure 1A, left panel). Furthermore, 5-LO-deficient mice exhibited enhanced expression of IL-12, IFN-γ, and nitrogen oxide synthase 2 in the lungs as compared with wild-type mice, indicating negative regulation of Th1 response by 5-LO during *M. tuberculosis* infections (66). Similar findings on the negative regulation of Th1 response by 5-LO were observed in *Brucella abortus* infections in mice (68). Activation of the 5-LO pathway was further demonstrated to impair host T cell immunity by preventing cross-presentation of *M. tuberculosis* antigen by dendritic cells (18). Interestingly, at the human population level, single nucleotide polymorphisms in eicosanoid receptor gene, EP2, and heterozygosity for leukotriene A4 hydrolase polymorphisms have been reported to modulate host susceptibility to tuberculosis, highlighting the critical roles of eicosanoids in mediating infections (69, 70).

Clearly, as exemplified by the multifaceted functions of eicosanoids, each lipid entity can be a man’s meat and another man’s poison. The type of eicosanoids and the fine balance between the pro-inflammatory and anti-inflammatory mediators are critical determinants of infection outcomes. Should this balance be disrupted, the host will be detrimentally affected either by high pathogen loads due to decreased clearance, or uncontrolled, non-specific inflammation (cytokine storms) leading to poor host outcomes (71). A more refined understanding of the functions of eicosanoids during interactions between specific pathogens and their hosts may potentially pave the future of host-directed therapy against these infections. In fact, a range of drugs (for instance, aspirin) that target eicosanoid biosynthesis are clinically available and can be further evaluated for their effects on bacterial infections.

### Glycerophospholipids

As one of the main constituents of the mammalian membrane bilayer, glycerophospholipids have been demonstrated to be used by pathogens to evade host defenses. Choline-containing glycerophospholipids such as phosphatidylcholine are predominantly localized to the outer membrane leaflet of eukaryotic cells, while amino-containing glycerophospholipids such as PS and phosphatidylethanolamine are predominantly maintained in the inner membrane leaflet (72). Redistribution of PS to the external surface of the plasma membrane is a key event during apoptosis and has been known as one of the emblematic signals leading to tagging of cells for efferocytosis, a phagocytic process for removal of dead cells (24, 73). This signaling pathway has been shown to be manipulated by *L. monocytogenes* to ensure their own survival in the infected host (74). The pore-forming toxin listeriolysin O from *L. monocytogenes* promotes the release of bacteria-containing protrusions from host cell membranes, generating PS-coated vesicles that mimic apoptotic cells. This subsequently promotes efferocytosis by binding to the PS-binding receptor TIM-4 expressed on uninfected macrophages, and eventually facilitates cell-to-cell spread in macrophages *in vitro* (74). While efferocytosis promotes the spread of infection by *L. monocytogenes*, this mechanism of cellular clearance appears to be protective to the host for other intracellular bacteria. Notably, efferocytosis has been shown to effectively restrict the growth of *M. tuberculosis* (75), as the bacteria-infected macrophages are engulfed and killed by uninfected macrophages following apoptosis (75) (Figure 1B, right panel). This process is also effective in limiting the growth of *M. marinum* in infected macrophages by neutrophils (76).

Phosphoinositides, which are phosphorylated forms of the membrane glycerophospholipid, phosphatidylinositol, are extensively characterized mediators of intracellular signaling and play critical roles during infection. Phosphoinositides can be phosphorylated at the hydroxyl residues at positions 3, 4, or 5 of the inositol ring to produce different phosphoinositide species. These include phosphatidylinositol 3-phosphate [PI(3)P], phosphatidylinositol 4,5-biphosphate, and phosphatidylinositol 3,4,5-triphosphate, which are involved in endocytosis and phagocytosis (77, 78). In fact, different phosphoinositide species exhibit distinct characteristic subcellular distribution patterns due to the organelle-specific phosphoinositide kinases and phosphatases (78). These phosphoinositide species interact with actin-binding proteins through recognition of its head group by PH, PX, ENTH, ANTH, or FYVE domains (20). Many pathogens are able to manipulate host phosphoinositides metabolism to trigger their uptake into macrophages or non-phagocytic cells (21). *M. tuberculosis* escape phagocytic killing by macrophages through blocking phagosomal maturation *via* interference of phosphatidylinositol 3-kinase [PI(3)K] signaling (22). *M. tuberculosis* lipoarabinomannan acts as a phosphatidylinositol analog and inhibits cytosolic calcium increase, leading to the blocking of the Ca^2+^/calmodulin PI(3)K hVPS34 cascade. This signaling pathway is needed to produce PI(3)P on liposomes or phagosomes and trigger downstream calmodulin kinase II-mediated EEA1 recruitment to phagosomal membranes, which are ultimately required for phagosome maturation (23). In addition, the lipid phosphatase, SapM, which is secreted by the bacterium, is responsible for the inhibition of phagosome-late endosome fusion by hydrolyzing PI(3)P and thus blocking phagosomal maturation (79). Concurrently, mycobacterial phosphatidylinositol mannoside stimulates early endosomal fusion by early recruitment of Rab5, which blocks the acquisition of late endosomal/lysosomal constituents (80) (Figure 1B, left panel). The orchestration of these intracellular signaling events mediated by lipids favors the intracellular persistence of *M. tuberculosis*, leading to chronic infections.

Similar to *M. tuberculosis, Salmonella* can infect macrophages and escape killing by these phagocytes to survive and replicate within host cells (81). More extensive research on salmonellosis has been carried out in non-immune epithelial cells since *Salmonella* initiates its infection *via* invasion of the intestinal epithelium. Invasion of host cells is dependent on two type III secretion systems (T3SSs) encoded on the SPI-1 and SPI-2 (82–84). One of the effector proteins of the T3SS of *Salmonella*, SopB (also known as SigD), is a phosphoinositide phosphatase which shows sequence homology to mammalian inositol polyphosphate 4-phosphatases (85) and type II inositol 5-phosphatase synaptojanin (86). The activity of SopB is required for invasion, formation, and maintenance of *Salmonella*-containing vacuoles (SCVs) in epithelial cells and macrophages (87, 88).

Manipulation of host glycerophospholipid metabolism by bacterial effector proteins is not unique to *Salmonella*. The *Legionella* Dot/Icm type IVB secretion system effector protein, VipD, is a phospholipase A1 which binds to and is activated by the endosomal regulator Rab5. The resultant removal of PI(3)P mediated by the bacterial phospholipase blocks endosomal fusion with *Legionella*-containing vacuoles, shielding the intracellular pathogen from the microbicidal endosomal compartment (89). Besides VipD, two other *Legionella* effectors, LecE and LpdA, which are localized to *Legionella*-containing vacuoles, are also capable of manipulating biosynthesis of host glycerophospholipids. Indeed, bacterial phospholipases are well-characterized virulence factors, notably the alpha-toxin from *Clostridium perfringens* (90). The function of phospholipase C from *M. tuberculosis*, on the other hand, remains less clear. Originally proposed to promote *M. tuberculosis* growth in the late stage of infection in mice (91), the role of phospholipase C in mycobacterial virulence has been recently challenged by the works of Le Chevalier and coworkers. In contrast to the works by Raynaud et al. which demonstrated a 1.5-log growth reduction in the phospholipase C mutant (91), the latter study did not detect significant differences between the wild-type and mutant bacteria (92). Clinical *M. tuberculosis* strains with interruptions of all four mycobacterial phospholipase genes have been isolated in patients with active tuberculosis, supporting a less crucial role of this enzyme in the infectious cycle *in vivo* (93).

While bacterial phospholipases can be detrimental to the host, conversely, host phospholipase activities can antagonize the survival of intracellular pathogens. In the context of mycobacterial infections, host phospholipase D and lysosomal phospholipase A2 have been implicated in the cells’ ability to control intracellular mycobacterial growth (94, 95). It should be noted that these studies were focused on the enzymes, and the exact lipid mediators remain to be identified. Nonetheless, the cumulative evidence of the involvement of both host and microbial glycerophospholipids metabolizing enzymes in the regulation of the infection process highlights the complexity of interplay of the metabolic networks of two organisms. This intricacy is not restricted to glycerophospholipids and will be reflected in the following subsections on two other major eukaryotic lipid classes, sphingolipids and sterols.

### Sphingolipids

Sphingolipids are another key component of eukaryotic cell membranes. They comprise of a long-chain amino alcohol (also known as a sphingoid base or long-chain base) to which a fatty acid can be covalently linked to form ceramide. Structural variants arise from head group substitutions, as well as chain length differences and hydroxylation of the sphingoid bases and fatty acyl chains, giving rise to tens of thousands of different molecular species with diverse functions (96–98). Sphingolipids are partners with cholesterol in eukaryotic membranes, forming specialized domains (commonly termed as lipid rafts) and serve as signaling platforms and/or entry sites during pathogen invasion. The functions of lipid rafts in host–pathogen interactions have been extensively reviewed (99–102).

Beyond their structural functions, sphingolipids also serve as signaling molecules which mediate the infection process (103, 104). Sphingosine-1-phosphate (S1P) is an active metabolite formed by sphingosine kinases 1 and 2, and are involved in immune cell trafficking, through engagement with G-protein-coupled receptors (S1PR 1–5). S1PRs are expressed on different immune cells, and macrophages mostly express S1PR1 and S1PR2 (105). Recently, it has also been shown that human alveolar macrophages express high levels of S1PR3 and S1PR4, and lower levels of S1RP5 (26). Expression of S1PRs determines the function of an immune cell, as they play critical roles in lymphocyte trafficking, differentiation and triggering of inflammatory responses (106). During mycobacterial infections, phagocytosis is uncoupled from cytosolic calcium level elevation, mediated by the inhibition of macrophage sphingosine kinase activity and consequently, the downregulation of S1P levels. The blockade of phagosome–lysosome fusion allows the intracellular bacterium to avoid the bactericidal phagolysosomes to continue to persist within host cells (107). Stimulation of macrophages with S1P leads to increased killing of internalized *Mycobacterium* species through the acidification of phagosomes *via* host phospholipase D (108) (Figure 1C, right panel). Additionally, S1P modulates mycobacterial infections by promoting antigen processing and presentation (109). These studies, taken together, highlight the antimycobacterial properties of the mediator lipid S1P.

The phagolysosomal compartment is clearly crucial for defense against infection with intracellular pathogens. Besides S1P, phagosomal maturation is also regulated by other sphingolipids. Host acid sphingomyelinase (ASMase), which generates the signaling molecule ceramide, is required for the proper fusion of late phagosomes with lysosomes (110). Delivery of ASMase to mycobacteria-containing phagosomes is regulated by sortilin, which requires interactions with adaptor protein AP-1 and monomeric gamma-ear-containing ADP ribosylation factor-binding proteins (GGAs) (111). Sortilin knockout mice are more susceptible to *M. tuberculosis* infections. Moreover, treatment of mouse macrophages with desipramine, an ASMase inhibitor, resulted in increased mycobacterial survival, indicating that ASMase is required for restricting the growth of *M. tuberculosis*. The crucial role of this host enzyme in controlling intracellular bacteria can be further illustrated by the increased susceptibility of ASMase knockout mice to *L. monocytogenes* infections (112). Functional ASMase is also involved in the bactericidal activity of macrophages against *S. enterica* serovar Typhimurium (25). In contrast to the protective effects of ASMase, neutral sphingomyelinase is associated with superoxide production during *M. bovis* BCG infections *in vitro* and *in vivo*. The superoxide produced during infection inhibits autophagy and therefore reduces bacterial clearance (113).

Various intracellular pathogens have also evolved metabolic strategies to hijack host sphingolipids to promote their pathogenicity. This is evident from the presence of sphingolipid metabolizing genes in bacterial genomes (114), although sphingolipids are synthesized only in eukaryotes (with few exceptions in prokaryotes, such as *Sphingomonas* species). *M. tuberculosis* encodes a novel outer membrane protein, Rv0888, which possesses potent sphingomyelinase activity (115). The bacterial protein has been shown to promote intracellular infection in macrophages *in vitro*, but is not required for virulence of *M. tuberculosis* in mice (115). While the functions of Rv0888 in mycobacterial infections remain to be clarified, it is interesting to note that a study conducted in as early as 1948 had already demonstrated that sphingomyelin supports the growth of the tubercle bacilli *in vitro* (116). Besides *M. tuberculosis*, a phospholipase C with sphingomyelinase activity has been characterized in *L. monocytogenes* (117). However, its function in *Listeria* infections has yet to be elucidated. By contrast, *L. pneumophila* produces an effector protein, *Lp*Spl, which shares structural and sequence homology to the eukaryotic counterpart sphingosine-1 phosphate lyase (118). Similar to the glycerophospholipid metabolizing enzymes, VipD, LecE, and LpdA, outlined above, *Lp*Spl is an effector protein of the Dot/Icm type IVB secretion system. It has been shown that *Lp*Spl activity prevents an increase of sphingosine levels in infected macrophages (Figure 1C, left panel). In addition, the bacterial sphingolipid metabolizing enzyme inhibits autophagy during macrophage infection and is required for efficient infection of mice. This represents a novel mechanism of inhibition of autophagy by an intracellular bacterium through perturbation of host sphingolipid biosynthesis. Strikingly, a similar strategy was observed in the facultative intracellular bacteria *B. pseudomallei* (119).

Evidently, the balance of host sphingolipids is another determinant of successful infections by intracellular bacteria. S1P is one of the most obvious sphingolipid metabolites which exhibit bactericidal activity. Various drugs which target the S1P axis are available and in fact used in clinical trials for various indications (120). With a deepened understanding of S1P and sphingolipids in inflammation and infection, the appropriate control of the S1P levels and potentially other sphingolipids may be targets for generation of novel drugs in treatment of various infections.

### Sterols

Sterols are major components of many animals (zoosterols) and plants (phytosterols), but only a few bacteria are able to synthesize sterols. In bacterial membranes, hopanoids, a class of pentacyclic triterpenoids, execute the functions of sterols (121). The most well-known zoosterol, cholesterol, plays critical roles in modulating cellular functions. These include regulation of membrane fluidity, phagocytosis, cell signaling, and formation of lipid rafts with glycosphingolipids (27, 122–124). In addition, cholesterol also serves as precursors for bile salts, steroids, and vitamins (125). Cholesterol in mammalian cells can be obtained from exogenous sources such as low-density lipoproteins or endogenously synthesized. Cholesterol accumulation is commonly observed in the form of lipid droplets in infected cells as well as in biopsies, such as in lepromatous leprosy tissues and tuberculosis granulomas (126, 127). At the cellular level, it has been demonstrated that some pathogens utilize cholesterol on lipid rafts for invasion and intracellular replication (99–102). Plasma membrane cholesterol plays an essential role for host cell uptake of *M. tuberculosis*. In addition, the association of cholesterol with the coronin 1 protein in phagosomal membranes ensures the intracellular survival of *Mycobacterium* in coronin 1-coated phagosomes by preventing degradation of the tubercle bacilli in lysosomes (128) (Figure 1D, left panel). The need for cholesterol in mycobacterial infections and survival is best emphasized by the presence of multiple genes involved in cholesterol transport and catabolism, although the bacterium does not synthesize cholesterol *de novo*. These genes include the cholesterol transporter, Mce4 and the transcriptional regulator KstR, which control the expression of bacterial genes involved in sterol catabolism (129–132). Furthermore, mutants lacking genes in cholesterol utilization fail to establish infection in macrophages (133).

Besides *Mycobacterium, Listeria* species and *Salmonella* species are also capable of utilizing host cholesterol to promote their survival in macrophages. The cholesterol-binding cytolysin, listeriolysin O, secreted by *L. monocytogenes* binds to cholesterol embedded in the lipid bilayer of eukaryotic cell cytoplasmic membranes to facilitate bacterial escape from the phagosomal compartment into host cell cytosol (134–136). Listeriolysin O also performs other functions, including modulation of inflammatory responses through activation of caspase-1, leading to IL-18 secretion from the infected macrophages (137). In fact, cholesterol-binding cytolysins are also produced by other pathogens, including perfringolysin O from *C. perfringens*, and facilitate bacterial escape from phagosomes and survival in macrophages (90). *Salmonella*, which reside in vacuoles, take a distinct approach. During *Salmonella* infections, cholesterol accumulates in the SCV (138). SseJ, another effector protein of the T3SS, has been reported to be involved in cholesterol esterification and stabilization of the SCV (139). Interestingly, the modulation of macrophage cholesterol levels with statins has been shown to augment host protection against various intracellular pathogens including *M. tuberculosis, M. leprae, S. enterica* serovar Typhimurium, and *L. monocytogenes* (28, 126, 140, 141).

While it has been extensively proven that cholesterol supports the survival of intracellular pathogens within infected macrophages, cholesterol can also exert protective effects on the host during infection by specific intracellular bacteria. The protective role of cholesterol for the host has been shown recently during infection by *Coxiella burnetii*, the causative agent for Q fever. Mulye et al. (142) reported that increased cholesterol levels in the parasitophorous vacuole by cholesterol supplementation or treatment with U18666A inhibited the growth of intracellular bacteria. Cholesterol induces acidification of the parasitophorous vacuole and eventually, killing of the intracellular bacteria (142). Although this study was performed on mouse embryonic fibroblasts, earlier works on *C. burnetii*-infected THP-1 macrophage-like cells had also demonstrated inhibition of intracellular *C. burnetii* growth by U18666A (143), suggesting that alterations in cholesterol levels can affect the survival of this pathogen in immune cells. The biosynthetic intermediates as well as metabolites of cholesterol can also improve resistance of the host cells to infection. During *Listeria* infection of macrophages, lanosterol, an intermediate of cholesterol biosynthesis, accumulates due to type I IFN-dependent histone deacetylase 1 transcriptional repression of lanosterol-14α-demethylase, the gene product of Cyp51A1. Besides the modulation of IFN-β-stimulated gene expression and the effects on cytokine production, accumulation of lanosterol also leads to an increase of membrane fluidity and ROS production, thus potentiating phagocytosis and the destruction of bacteria (144).

Cholesterol also serves as a precursor for vitamins, including the fat-soluble vitamin D. Interestingly, vitamin D can have protective effects against bacterial infections (145). In the context of mycobacterial infections, 1,25-dihydroxy-vitamin D_3_ (1,25D), the active metabolite of vitamin D, promotes maturation and activation of human monocytes and macrophages and reduces bacillary replication. In a macrophage–epithelial cell coculture system, 1,25D enhanced IL-1β expression and induced secretion of the cytokine from *M. tuberculosis*-infected macrophages *via* the NLRP3/caspase-1 inflammasome pathway. IL-1β secreted from macrophages reduced mycobacterial burden by stimulating the epithelial production of the antimicrobial peptide DEFB4/HBD2. This suggests that the control of *M. tuberculosis* infection by vitamin D is modulated by macrophage–epithelial paracrine signaling (146) (Figure 1D, right panel). In fact, vitamin D deficiency and vitamin D receptor polymorphism (147) have shown to affect human susceptibility to tuberculosis, and vitamin D-based oxysterols can promote clinical improvements in tuberculosis patients (148). Interestingly, the vitamin D metabolite 25(OH)D_3_ has also been found to act synergistically with the bacteriostatic antituberculosis drug phenylbutyrate, providing a potential indication of adjunct therapy for tuberculosis through resolution of inflammation and enhancement of bacterial clearance (149).

The possibility of using vitamin D, and potentially sunlight, to fortify antituberculosis resistance is an exciting and straightforward approach for tuberculosis interventions. Definitely, it will be of interest to extend such studies of vitamin D to other microbial infections. However, specifically for cholesterol, with its mixed roles of being both protective and harmful to the host during infection, as well as its central roles in diverse cellular processes, targeting cholesterol metabolism alone may cause undesired complications. Combination treatment or targeting of specific downstream products of cholesterol-induced signaling may be explored as an alternative.

## Microbial Lipids—Manipulators of Macrophage Metabolism

### Glycolipids—Lipopolysaccharides (LPS)

Besides protein-based bacterial virulence factors which are capable of manipulating host lipid metabolism during infection, foreign lipid structures expressed on bacteria surfaces can also act as pathogen-associated molecular patterns which induce immune responses as well as remodeling of lipid metabolism in macrophages. LPS, a major component of the outer membrane of Gram-negative bacteria, is made up of a conserved lipid A, core oligosaccharide regions and a long polysaccharide chain with variable carbohydrate subunits (150). LPS is well known as a ligand for toll-like receptor 4 (TLR4) (151–153) and toll-like receptor 2 (TLR2) (154, 155). Signals triggered by TLR4 upon activation by LPS have been extensively studied and reviewed (151, 156–158). Briefly, this signaling cascade is initiated by LPS binding protein which binds directly with LPS and is transferred to CD14. This then dissociates LPS aggregates into monomeric forms and presents them to the TLR4–MD-2 complex. Binding of LPS to TLR4–MD-2 complex eventually activates NF-κB and IRF3, leading to the production of pro-inflammatory cytokines (*via* MyD88-dependent pathway) or type-1 interferon (MyD88-independent pathway) (159). In 2013, two independent studies demonstrated that LPS activates inflammasomes *via* murine caspase-11, independent of TLR4 (160, 161). Following that, Shi and coworkers showed that human caspase-4 and -5 [orthologs of murine caspase-11 (162)] served as intracellular sensors for LPS and lipid A. The binding of LPS to caspase-4 leads to pyroptosis in human monocytes and non-immune cells (163). Due to its ability to trigger strong immune responses, LPS has always been linked to a variety of pathologies such as septic shock and death, while inhibition of LPS-induced signaling pathways often favors the hosts (161, 164–166).

The impact of LPS on host lipid metabolism has been fairly well characterized. Very early studies demonstrated that LPS administration produced hypertriglyceridemia, with increased lipoprotein production and decreased lipoprotein clearance (167, 168). The effect of lipogenesis and lipolysis seemed to be affected by the dosage of LPS, as Feingold and coworkers showed that low doses (10 ng/100 g body weight) induced hepatic secretion of triglyceride, while high doses (50 μg/100 g body weight) decreased the clearance of triglyceride-rich lipoprotein (169). LPS also increased lipid body formation in human macrophages, through increasing the fatty acid uptake and reducing lipolysis, hence increasing triglyceride retention in the cells (170). Furthermore, turnover of glycerophospholipids during LPS stimulation has been observed, which is mediated by induction of acyl-CoA synthetase 1 (171). Accumulation of lipid droplets in non-adipocytic cells has also been reported as a pathological feature in infectious diseases, and it has been demonstrated to be an important site for PGE_2_ synthesis (172, 173). Lipid body formation is largely dependent on TLRs, especially TLR2 and TLR4, as TLR2-deficient and TLR4-mutated mice both failed to form lipid droplets triggered by LPS (172, 173). When induced by LPS, lipid droplets are formed *via* the p38 α/β and PI(3)K/Akt pathways (174), and drugs blocking these pathways have been proven to inhibit their formation (174, 175).

A combined lipidomics and transcriptomics study on mouse macrophages upon LPS stimulation by Dennis and coworkers has brought together a system-based overview of the temporal and subcellular dynamics of macrophage lipid remodeling elicited by LPS (176). Treating RAW264.7 cells with Kdo_2_-lipid A, a chemically defined substructure of LPS, increased COX-2 related lipid metabolites immediately after the stimulation, followed by sterols, sphingolipids, glycerophospholipids, and glycerolipids (176). Based on the reported findings on LPS-induced changes in sphingolipids, KÖberlin and coworkers recently examined the lipid metabolic network of RAW264.7 cells during TLR signaling and showed that genes involving sphingolipids metabolism are tightly modulated upon TLR4 and TLR9 stimulation. Intriguingly, this study revealed that innate immune responses are modulated by a circular network co-regulating sphingolipids and glycerophospholipids (177). These studies have contributed to our current understanding on the complexity of the macrophage lipid metabolic networks during inflammation, and it will be of interest to identify novel small molecules which can target lipid metabolism for immune protection.

### Glycolipids—Trehalose-6,6′-Dimycolate (TDM)

Besides the Gram-negative bacteria-specific LPS, lipids produced by other bacteria also possess host immune- and metabolism-modulating functions. *M. tuberculosis* and *M. leprae* are notorious for their fat-loving nature as well as their cell walls which are dominated by lipids (178, 179). TDM, also known as cord factor, is a glycolipid abundantly expressed on the cell wall of mycolic acid-containing bacteria such as *Mycobacterium, Nocardia, Tsukamurella, Gordona, Rhodococcus*, and *Corynebacterium* species and has previously been identified as a virulence factor (180). The TDM molecule consists of sugar trehalose esterified to two mycolic acid residues, and the length range of residues varies from 20 to 80 carbons depending on the bacterial species. TDM is responsible for the characteristic colony morphology observed in virulent *M. tuberculosis* due to its hydrophobic nature (181). In addition, at the gene expression level, TDM from *M. tuberculosis* H37Rv upregulates multiple cytokines (TNF-α, IL-1β, IL-1m, IL-10, and MCSF-1) and chemokines (CCL3, CCL4, CCL7, CCL12, CCR12, CXCL1, CXCL2, and CXCL10) (182), proving the ability of TDM to initiate the macrophage immune responses *in vitro* (183). Strikingly, TDM alone is sufficient to induce activated foreign body- and hypersensitivity-type granulomas in mice, as well as extensive foam cell formation, illustrating its importance in pathogenesis (127, 184–186). TDM also promotes *Mycobacterium* survival *in vivo* by inhibiting phagosome–lysosome fusion and protects the bacteria from destruction within macrophages (187, 188).

Besides its effects on the immune functions of macrophages, TDM has been shown to induce formation of caseating granulomas and foamy macrophages in the absence of *Mycobacterium* itself (127). The toxicity of TDM is greatly dependent on its surface crystalline structure. This can be enhanced by the presence of oil, as administration of TDM in oil-in-water emulsion has shown to induce higher mortality rates in mice, whereas administration of TDM without oil has no effect (189–193). Interestingly, *M. tuberculosis* preferentially associates with the lipid droplets in pulmonary lesions to increase the toxicity of TDM and initiation of necrosis (192). Separately, free mycolic acids have also been shown to induce macrophage lipid droplet formation (194). Keto-mycolic acid, the oxygenated form of mycolic acid, was found to transactivate the host orphan lipid-sensing nuclear receptor, testicular receptor 4 (TR4), and induce foamy macrophage formation *in vitro* and *in vivo* (195). TR4 is also involved in the polarization of macrophages toward a less microbicidal and an immunomodulatory M2 phenotype, which aids in the survival of *M. tuberculosis*. With more in-depth understanding of microbial lipid–host receptor interactions, guided designs of structural analogs of lipids to interfere with ligand–receptor binding holds promise as a combinatorial adjunct therapy.

Another mycobacterial lipid virulence factor that is capable of moderating macrophage functions, but differs in function from TDM, is phthiocerol dimycocerosates (PDIM) (196–199). PDIM has been proven to mediate phagosomal escape of *M. tuberculosis* by enhancing the membrane permeabilizing activity of ESAT-6, increasing phagosomal membrane destabilization and subsequent induction of apoptosis (200, 201). While PDIM and ESAT-6 are known to cause phagosomal membrane rupture, whether this process involves alterations in host lipid metabolism requires further investigation. Probing changes in host lipid metabolism is increasingly possible with the availability of a range of techniques, which we will cover in the next section.

## Probing Lipid Dynamics During the Host–Microbe Relationship

Despite the appreciation of the important roles lipids play in infectious diseases, it is not as well studied as compared with genes and proteins, due to chemical complexity of lipids and the limited availability of tools required. Most of our knowledge on the roles of lipids in the intimate host–pathogen relationship has been gained through genetic and cellular investigations, and only in recent years have sensitive and high resolution technologies for lipid analyses become available for revelation of novel insights of lipid functions during infections.

Traditional methods of lipid analysis include enzyme immunoassays (EIAs) and thin layer chromatography (TLC). EIAs, an established method particularly for cytokine and chemokine measurements in both infection and immunity research, have been commonly used to monitor the levels of various eicosanoids (202, 203). The underlying mechanism of EIA lies in the immunocomplexes formed between the lipids of interest and their antibodies. These assays serve as a fairly rapid method for lipid analyses, provided that the lipids of interest are well defined. Another restriction of EIAs is its limited availability, because lipids generally have poor immunogenicity, and the generation of high affinity anti-lipid antibodies is challenging using traditional hybridoma techniques (204). Furthermore, these assays have limited resolution (i.e., may not be able to separate between lipid molecular species) and sensitivity, and hence are not well suited for very low abundant signaling molecules. The use of TLC for the separation and detection of lipids have been described as early as the 1960s (205) and continues to be commonly used (127, 206, 207). Despite its low sensitivity and resolution, TLC offers the possibility to study the turnover of lipids and capture the temporal dynamics of lipid remodeling when used with radioisotopes labeling (46, 47).

**Figure 2:**
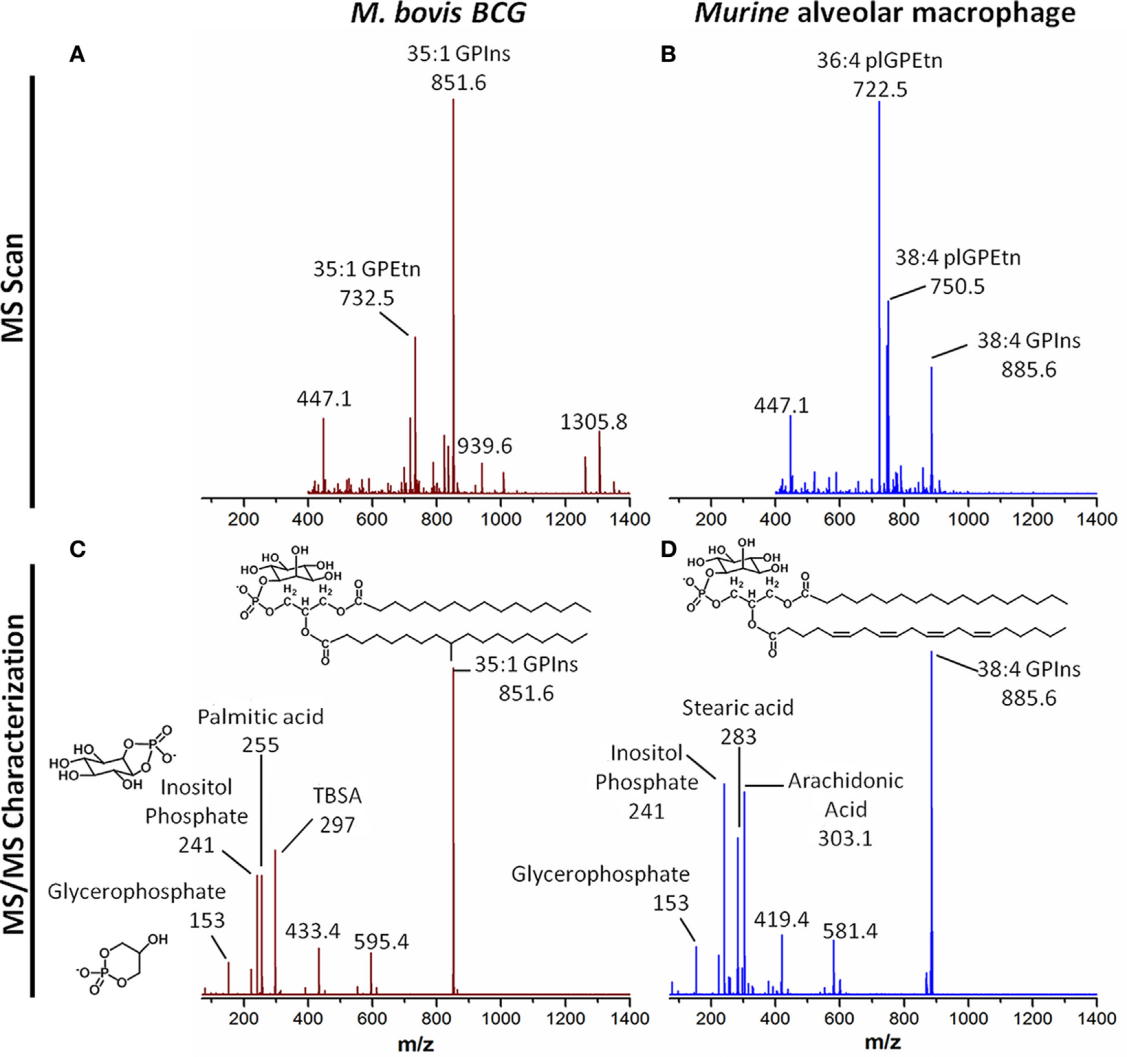
Resolving host and microbial (polar) lipid molecular species by mass spectrometry (MS), a tool for probing host–pathogen interactions. With advances in technologies particularly MS, it is now possible to profile, characterize, and quantify lipids from both microbes and macrophages as well as other host cells and tissues. **(A)** Single stage MS scan to generate the “lipid profiles” of *M. bovis* BCG, an experimental surrogate for *M. tuberculosis*. **(B)** Single stage MS scan to generate the “lipid profiles” of murine alveolar macrophages. **(C)** MS/MS of the major phosphatidylinositol from *M. bovis* BCG, with mass-to-charge ratio (*m*/*z*) of 851.6. Collision-induced dissociation reveals the ion contains palmitic acid (*m*/*z* 255) and TBSA (*m*/*z* 297), as well as the phosphoinositol (*m*/*z* 241) and glycerophosphate (*m*/*z* 153) headgroups. This confirms the sum composition of fatty acyls to be 35:1 (35 carbons, 1 double bond). **(D)** MS/MS of the major phosphatidylinositol from murine alveolar macrophages, with *m*/*z* of 885.6. Collision-induced dissociation reveals the ion contains stearic acid (*m*/*z* 283) and arachidonic acid (*m*/*z* 303.1), as well as the phosphoinositol (*m*/*z* 241) and glycerophosphate (*m*/*z* 153) headgroups. This confirms the sum composite of fatty acyls to be 38:4 (38 carbons, 4 double bonds). The bioanalytical tool can be applied to probe the dynamic changes in levels of thousands of species of lipids from both the host and the pathogens, leading to the identification of novel pathways involved in infection and inflammation. Abbreviations: GPEtn, phosphatidylethanolamine; GPIns, phosphatidylinositol; MS, mass spectrometry; MS/MS, tandem mass spectrometry; *m*/*z*: mass-to-charge ratio; plGPEtn, plasmalogen phosphatidylethanolamine; TBSA, tuberculostearic acid.

Recently, more advanced methods including gas chromatography–mass spectrometry, liquid chromatography–mass spectrometry (LC–MS), nuclear magnetic resonance (NMR) spectroscopy, and mass spectrometry (MS)-based imaging techniques have revolutionized the fields of metabolomics and lipidomics. These technologies allow for more efficient analysis of the diverse metabolites and lipids in biological samples, with deeper and more sensitive coverage at the lipid molecular species level. Notably, the LC–MS analyses of the monocyte/macrophage lipidome spanning membrane lipids (sterols, glycerophospholipids, and sphingolipids), and mediator and signaling lipids (phosphoinositides, eicosanoids, and phosphorylated sphingoid bases), have contributed to the revelation of the complex metabolic networks during macrophage differentiation (37, 38), TLR activation and signaling (176, 177, 208) as well as the complex host–pathogen interactions (8, 118, 209, 210). In the last 10–15 years, the lipidomes of various other host cell types including epithelial cells (211), platelets (212), and pathogens, including *M. tuberculosis* (213–215), *Clostridium novyi* (216), *E. coli* (217), *Enterococcus faecalis* (218), and *Pseudomonas aeruginosa* (219) have been established. The possibility to analyze both host and microbial lipids with high resolution structural information (Figure 2) has opened up new avenues to study the dynamics of lipid metabolism during the complex host–pathogen relationship.

While LC–MS- and NMR-based lipidomics approaches are extremely powerful, they lack the spatial resolution needed for determining the localization of lipids during the infection process (220), and, importantly, the source of the metabolites in the host–pathogen relationship. MS-based imaging has now offered a new dimension by providing spatial resolution of the metabolites during the infection process by various pathogens (221–223). Alternative novel imaging techniques involve the use of immunofluorescent lipids, isotopic labeling, or lipid-binding probes and microscopy for the spatial resolution and real-time analysis of the host–pathogen interaction, providing extensive information regarding the roles of lipids in pathogenesis and the immune responses. In the study by Barisch and Soldati, pulse-chase experiments using fluorophore-labeled compounds coupled with electron microscopy has provided novel insights about the translocation of host-derived fatty acids in *M. marinum* to serve as an energy source for the bacteria (224, 225). Metabolic labeling of lipid droplets has also been used for the exploration of the carbon flux within pathogen metabolic networks (47). The lipid-binding properties of bacterial toxins, including perfringolysin O and aerolysin, have also been harnessed as tools to probe lipid localization and signaling (226, 227). In recent years, the specificities of lipid probes have vastly improved, with the recent possibility to study single lipid species in living cells (228).

The current availability and upcoming developments in technologies which provide temporal and/or spatial information of lipids at the single lipid species level are extremely powerful, especially when used in combination with cell and chemical biology, genetics and other systems biology approaches, including proteomics and genomics. Together, these will provide new insights into the complexity of host–pathogen interactions, disease pathogenesis as well as identification of novel markers of inflammation and infection.

## Conclusion

Pathogenic infection of macrophages is an intricate process involving numerous sequences of events, during which lipids are clearly instrumental players. Each pathogen has evolved its own strategy to thrive in or kill the host cells, and it is not surprising to find that a single lipid class or species can display contrasting functions during bacterial infections, depending on the bacterial species and even cell types. A single lipid, such as cholesterol, can be utilized by a specific pathogen to promote its own survival in the macrophage and on the other hand, it can also be used by the host to assist in clearance of another bacterial species, making lipids a double-edge sword in the complex host–pathogen relationship. Adding on to the complexity is alterations in the fine chemistry of lipids. For instance, the oxidation of fatty acids can switch its function from being benign to bioactive, or from acting as the protagonist to the antagonist (and *vice versa*) in this complex interplay between host and pathogen. While we have described in this review how different lipid classes are involved in the infection process, it should be stressed that the system acts as a whole, and multiple components, including different lipid classes and proteins, act in concert to achieve the outcome of an infection. Hence, it is critical to undertake systems-level approaches to dissect the host–pathogen networks. Such in-depth analyses of the complex and interconnected lipid metabolic networks in infection and immunity will contribute to identification of potential lipids or metabolic pathways which can be potentially developed into therapeutics for treatment of infectious diseases or for boosting of immune functions.

## Author Contributions

OT, CKEA, and XLG contributed to the writing and review of the manuscript.

## Conflict of Interest Statement

The authors declare that the research was conducted in the absence of any commercial or financial relationships that could be construed as a potential conflict of interest.
